# Recycling Carbon Fiber from Carbon Fiber-Reinforced Polymer and Its Reuse in Photocatalysis: A Review

**DOI:** 10.3390/polym15010170

**Published:** 2022-12-29

**Authors:** Jie Wu, Xing Gao, Yueting Wu, Yutong Wang, Tat Thang Nguyen, Minghui Guo

**Affiliations:** 1 Post-Doctoral Mobile Research Station, Graduate School, College of Sports and Human Sciences, Harbin Sport University, Harbin 150008, China; 2 College of Wood Industry and Interior Design, Vietnam National University of Forestry Xuan Mai, Hanoi 13417, Vietnam; 3 Key Laboratory of Bio-Based Material Science and Technology of Ministry of Education, College of Material Science and Engineering, Northeast Forestry University, Harbin 150040, China

**Keywords:** CFRP waste, recycling methods, recycled carbon fiber, reuse, recyclable photocatalyst

## Abstract

Driven by various environmental and economic factors, it is emerging to adopt an efficient and sustainable strategy to recycle carbon fibers (rCFs) from carbon fiber-reinforced polymer (CFRP) wastes and reuse them in high-value applications. This review summarized the latest progress of CFRP waste recycling methods (including mechanical, chemical, and thermal methods), discussed their advantages and disadvantages, influence parameters and possible environmental effects, and their potential effects on the mechanical and surface chemical properties of rCFs. In addition, the latest optimization schemes of leading recycling technologies were detailed. According to the literature, CFs are the key points in the structural support of semiconductor-based recyclable photocatalytic systems and the enhancement of performance, which means that rCFs have high reuse potential in sustainable photocatalysis. Therefore, this paper also emphasized the possibility and potential value of reusing recovered fibers for developing recyclable photocatalytic products, which may be a new way of reuse in environmental purification often ignored by researchers and decision-makers in the field of CFs.

## 1. Introduction

In industrial applications, carbon fiber reinforced polymers (CFRPs) are composite materials formed using carbon fibers (CFs) in different forms as reinforcement and resins, ceramics, metals, cement, carbonaceous, polymers, or rubber as the matrix [1]. Compared to conventional materials, CFRPs have significant mechanical, thermophysical, and thermal ablation properties, including high strength ratio, high stiffness, lightweight, excellent wear resistance, corrosion resistance, durability, and thermal ablation resistance [2]. For example, the tensile strength of CF-reinforced resins could usually be higher than 3500 MPa, 7–9 times that of steel, and reach a tensile modulus of 230–430 GPa, much higher than steel and aluminum alloys. In addition, the specific strength of CFRPs could reach over 2000 MPa/cm^3^ compared to 59 MPa/cm^3^ for A3 steel. As a result, CFRPs have been the leading contenders in various high-value and high-performance applications, from aerospace to ground transportation to sporting goods and medical applications [3,4,5].

**Figure 1:**
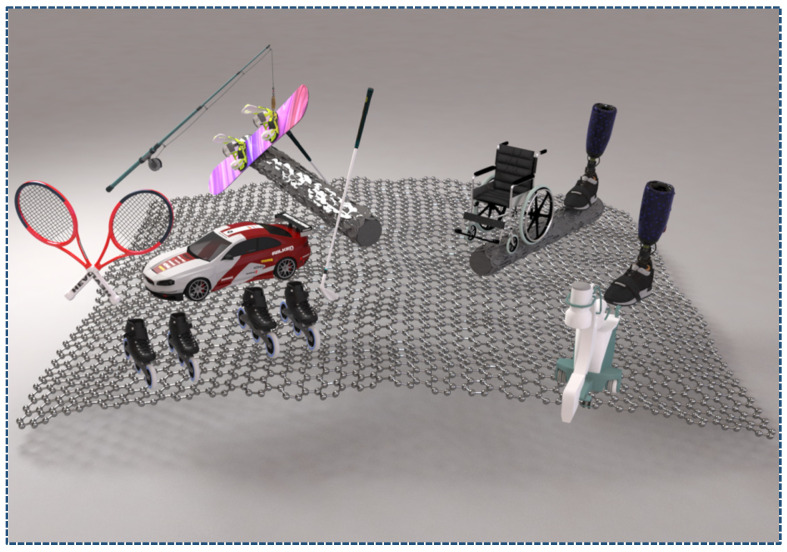
Various sports goods and sports medical devices obtained from CFRPs.

In recent years, CFRPs have expanded into more mass-oriented market segments, including the sports industry [6]. Mechanical properties such as strength, toughness, and elasticity are important aspects of sports equipment to meet market demand [7,8]. CFRPs (such as CF-reinforced epoxy resins, plastics, rubber, and vinyl polymers) are widely used in the manufacture of sporting goods, such as fishing rods, golf equipment, rackets, snowboards, speed skates, and racing cars, replacing traditional metal components to improve the properties of sports equipment [9]. In sports medicine, CFRPs have also been used to develop and manufacture medical devices such as X-rays, limb prostheses, and wheelchairs since the late 1970s, taking advantage of their excellent radioactivity, light transmission, and mechanical properties. In addition, CFs possess the necessary properties of biomaterials, such as good flexibility, biocompatibility, long-term stability in vivo, and easy integration with conventional biomaterials. As a result, CF-reinforced tendons, spinal fixators, and other medical devices containing CFs are now widely used in clinical applications [10,11]. Figure 1 presents a variety of sporting goods and sports medical devices obtained from CFRPs.

According to statistics, the global demand for CFs and CFRPs in various applications is expected to be 117 and 194 kilotonnes in 2022 [1,12]. Meanwhile, the CFRP market is expanding significantly, with market growth of approximately USD 48.7 billion [13]. A direct consequence is the massive accumulation of associated waste, mainly generated from the manufacturing process (approximately 30–40 wt% of the total material) and end-of-life products [14,15]. Dry carbon fibers are a common CF waste in the manufacturing phase (approx. 40% of total CF waste), mainly from offcuts, bobbin ends, and sometimes selvage [16,17]. However, as they are not yet embedded in any polymer matrix, they have similar properties to unmodified/virgin carbon fibers (vCFs) [16,18,19]. They will not be discussed in detail in this work. In addition to dry carbon fibers, another common waste material are CFRPs. Global CFRP waste is expected to reach 20 kilotonnes per year by 2025 [14]. Among them, retired commercial aircraft are expected to produce more than 17,000 tons of CFRP waste, which is expected to increase since the aerospace industry has expanded the applications of CFRPs. Recycling end-of-life CFRP waste from aircraft (such as Airbus A350 and Boeing 787 Dreamliner) will be a critical challenge for the renewable energy sector in the future. For example, Airbus’ targets include recycling 95% of CFRP manufacturing process wastes through the recycling channel, and recycling 5% of the waste products back to the aerospace sector. To support the development of the recycled carbon fiber (rCF) market, technology demonstrators (e.g., rCF seatback demonstrators for aircraft seatbacks) have determined the feasibility of the CFRP recycling from retired aircraft and manufacturing rCFs-based composite material for aerospace applications [20].

**Figure 2:**
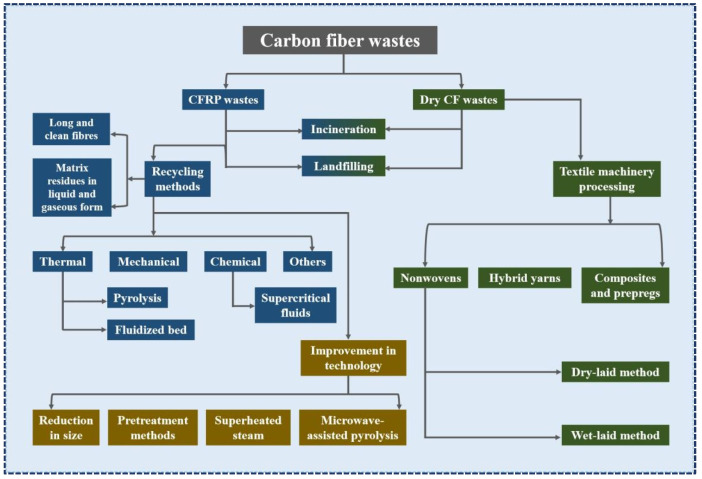
The main recycling management strategies for CFRP wastes and dry CF wastes, Data source: [1,16].

The environmental, economic, and policy requirements are prompting researchers to explore various routes for recycling CFRP and dry CF wastes [21,22,23,24,25]. Figure 2 shows the main recycling strategies for CFRP and dry CF wastes. To date, incineration and landfills have been the main methods of disposing of CFRP wastes. However, these routes are not feasible, especially as they release large amounts of pollutants into the environment and lose all high-value resources [26,27,28]. Life cycle assessment analyses show that the production of vCFs is also expensive and energy-intensive, with a serious negative impact on the environment [29]. The cost and benefit analysis indicates that obtaining rCFs is much cheaper than vCFs, which is considered one of the main advantages of recycling CFRP wastes. The economic evaluation should consider various cost factors involved in the recycling chain, such as waste collection, labor, transportation, pretreatment, recycling, and remanufacturing. Regardless of the method of recovery of energy and materials from waste processing, compared with other recycling methods, incineration or landfill have the lowest operating costs. Among the different recycling methods for CFRP wastes, the mechanical method is the cheapest, while the supercritical water or pyrolysis methods have a higher operation cost. In addition, researchers have found that the energy demand of the microwave-assisted pyrolysis method is one-third of that of the traditional pyrolysis method; thus, the operation cost is significantly reduced [16].

The adequate recycling of CFRPs, including the recycling of polymer matrices and rCFs, could bring some major challenges. Firstly, the recycled process is complicated. For thermoset polymer matrix composites (represent around 80% of the CFRP market) [30], due to covalent intermolecular chemically cross-linked polymer chains, thermoset matrixes have superior stability, mechanical properties, and fiber-matrix adhesion, which cannot be melted, remolded and recycled [31,32,33,34]. Secondly, CFs are the most expensive part of CFRPs, so how can the polymer be stripped to recycle the embedded rCFs? A variety of recycling techniques have been developed, such as the mechanical recycling method, chemical recycling method, and thermal recycling method [1,35,36,37,38,39]. The investigations regarding the main recycling methods in recent years are enlisted in Table 1.

Although the mechanical recycling method is cost-effective and can be applied on an industrial scale, rCFs have weaker mechanical (low strength) and physical properties (size reduction). Therefore this technology is not suitable for high-load structural applications [54]. However, chemical recycling methods can produce rCFs with clean and smooth surfaces and retain the mechanical properties of vCFs. However, it is difficult to promote commercially due to the high cost of solvents and equipment, the harsh conditions, and the numerous hazardous gases and waste streams generated [21]. To recycle CFRPs more efficiently, thermal recycling methods such as pyrolysis, steam, and fluidized bed methods are commonly employed [48,55,56]. This field relies on chemicals in the supercritical phase to dissolve the polymer matrix, and the tensile strength of rCFs can reach 80–95% of vCFs. However, the coke as-formed during the heat treatment tends to deposit on the surfaces of the rCFs [57].

The real transformation of rCFs (including dry carbon fibers) into a high-value resource is likely to be important research for the future development of CFs and CFRPs. Therefore, it is necessary to develop sustainable recycling systems. Developing reuse strategies for rCFs is part of ongoing research, such as spinning into yarns, manufacturing nonwovens and non-crimp prepreg fabrics using combined rCFs and vCFs, and new composite materials [16,58]. Interestingly, the comprehensive performance advantages of rCFs make them have innovative reuse values in photocatalysis. For example, the excellent adsorption properties are conducive to adsorbing molecules. On the other hand, excellent electrical conductivity is conducive to charge transport; high porosity facilitates the flow of water or gas; the high flexibility, excellent mechanical properties, and ease of recycling indicate that these materials are stable, flexible, and recyclable [59,60,61]. Therefore, rCFs can act as carriers and fabricate photocatalytic materials with easy separation by fixing semiconductor nanoparticles on their surfaces. This reuse strategy of rCFs can overcome the limitation that the powder photocatalyst is not easy to recycle, increasing the potential for large-scale application in the photocatalytic degradation of pollutants. However, it is still a new exploration idea, and no relevant research report has been found.

In summary, rCFs are derived from two main sources: (1) dry carbon fiber wastes with similar properties to vCFs, and (2) recycled CFRP wastes. However, people still have a limited understanding of the reuse of rCFs (an important link in the CF waste treatment chain) and the value-added products obtained, but few investigations can be found. Notably, in environmental protection and life cycle assessment, the fabrication of some new photocatalytic materials leaves research space for the reuse strategies of rCFs. Therefore, this work mainly focuses on some important developments in CFRP waste treatment and puts forward new views on the reuse pathways of rCFs. Firstly, the recent advances in different recycling methods of CFRP wastes were reviewed comprehensively, including their advantages and disadvantages, influence parameters, and possible environmental influences. Secondly, some of the latest optimization strategies in recycling methods were summarised, which can provide a more comprehensive understanding of the CFRP recycling industry. Finally, this work provides an innovative review of recent reports on the use of CF materials to fabricate photocatalytic products for the degradation of pollutants in the environment, opening up new possibilities for developing the reuse of rCFs and value-added products for rCFs. This reuse strategy of rCFs is rarely noticed and unreported. Therefore, this work aims to fill this research gap.

## 2. Methods of Recycling rCFs from CFRPs

**Figure 3:**
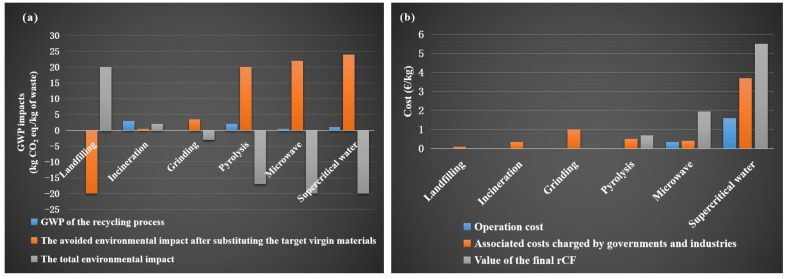
Comparison of (**a**) environmental and (**b**) economic impacts of various waste management and recovery methods, Data source: [16].

Over the last decade, several studies have been reported on CFRP waste recycling techniques, including mechanical, chemical, and thermal recycling methods (as shown in Figure 2 and Table 1). In the long term, efficient and sustainable solutions can minimize the environmental (global warming potential, GWP) and economic impacts of CF waste accumulation (Figure 3) and create new high-value-added products [16,62,63]. The recycling of CFRPs depends largely on whether their matrix type is thermoplastic or thermosetting [64]. The former can be used to manufacture new products by remelting and reshaping [64,65]. The latter focuses on releasing embedded rCFs by depolymerizing the matrix into a complex mixture of gaseous, liquid, and solid chemicals using a new process technology [19,64,65]. This section will summarise these contents and the aspects, such as the mechanical properties of rCFs obtained from various recycling methods. This will provide some insight into finding a suitable recycling process.

### 2.1. Machining Recycling Method

Mechanical recycling techniques usually use multi-shaft shredders and cutters, which primarily cut, chop, shatter and grind composite parts into small pieces, followed by sieving to extract powder and fiber materials. Papaspyrides et al. [36] studied the feasibility of recycling rCFs from thermosetting composites to produce new composite materials and found some positive results. The rCFs with acceptable quality and quantity could be obtained from CF/epoxy resin by a quick and simple grinding process, which could be reused in new-generation thermoplastic composites. The tensile tests showed that these new materials performed well compared to the samples using vCFs. Thomas et al. [66] mechanically recycled the CF powder with a length of 1.25 mm from composite laminates and added it to the resin (20 wt%). The results showed that the compressive strength, flexural strength, and modulus of elasticity could be enhanced by 20%, 30%, and 30%, respectively. Colucci et al. [67] filled polyamide 66 (PA66) with short rCFs to produce a new thermoplastic composite and then mechanically recycled it, including pelletizing, remelting, and re-injection molding. It was found that although the length of the rCFs was short, the effect on the mechanical properties of the re-manufactured parts was negligible.

Unlike landfills and incineration, mechanical recycling methods can produce other raw materials. They are usually fast in processing, easy to expand, and do not produce CO or CO_2_ and other waste gases. Still, they need to consume a lot of energy in the recycling process (e.g., approximately 200 MJ/kg of electrical energy) [68,69]. Another challenge of mechanical recycling methods is the production of rCFs with resin residues on their surfaces and considerable loss of mechanical properties, including shortened lengths and uneven surface properties, which reduces their reusability in new CFRP products. Such rCFs are mainly used in low-value applications, such as fillers for construction materials (concrete, asphalt, or coatings) [19]. However, they have no potential market for development, thus leading to a significant loss of economic value. Applications and research in this area have rarely been developed as mechanical recycling methods are considered an uneconomic downward cycle. Currently, institutions related to applying mechanical recycling include Procotex Fibre Processing (Belgium) and the University of Manchester (UK) [1]. In a word, all composite materials can be recycled through mechanical recycling technology. However, it is not easy to obtain rCFs that can match the performance of vCFs.

### 2.2. Chemical Recycling Method

Developing chemical recycling methods has already motivated some researchers to produce rCFs with essentially unchanged mechanical and physical properties [44,51,69,70]. Different types of reactive solvents can decompose the polymer matrix into soluble low molecular weight products, the end products of which will be fibers, inorganic fillers, and dissolved depolymerized resins and monomers. To increase the specific surface area in contact with the solution and promote matrix dissolution, the CFRPs are first mechanically crushed. The derived rCFs are washed at the end of the recycling process to remove residues from their surfaces [1,71], resulting in the rCFs with longer lengths and preserved mechanical properties. However, the hazards and toxicity of chemical solvents could have adverse effects on the environment [1,17].

Many studies have used subcritical or supercritical solvents to reduce these environmental issues to replace these chemicals at different temperatures and pressures, such as water, alcohol, ammonia, and organic solvents. The chemical recycling method of supercritical fluids is also known as solvent decomposition. Supercritical fluids have unique physical properties, including low viscosity, high density, good flow, mass transfer, heat transfer, and special solubility parameters [45,69,72]. In addition, they could be sensitive to changes in temperature and pressure. Removal of resin by solvent decomposition involves several steps [44]: (1) solvent diffusion; (2) reaction of the fluid with the fiber surface; (3) matrix dissolves into the fluid, and (4) mass transfer by convection. This chemical recycling method allows rapid and selective decomposition of CFRPs to obtain clean rCFs without significantly losing their mechanical properties. However, the operating conditions for this technology are harsh and costly. In addition, the theoretical research on supercritical fluid technology is not in-depth, so it has not been commercialized.

The chemical recycling method’s important parameters include solvent type, reaction time, processing temperature, and catalyst concentration. The processing efficiency of water and alcohols has been widely studied in different fluids. Due to the decrease in the dielectric constant of water molecules under supercritical conditions, water can be used as a non-polar solvent for dissolving organic compounds [45]. The critical point conditions for alcohols (200–300 °C, 2.0–6.0 MPa) are lower than those for water (374 °C, 22.1 MPa). Therefore, it is easier to recycle the process using alcohol. Piñero-Hernanz et al. [43] applied chemical recycling methods to recycle rCFs from CF-reinforced epoxy resins using various supercritical alcohols. Compared to vCFs, the rCFs with no matrix residues on their surfaces retained 85–99% mechanical strength. However, considering the cost-efficiency and environmental impact, water was mainly chosen as the fluid. Kim et al. [45] used supercritical water to remove 99.5% of the resin without a catalyst. The rCFs obtained were blended with cyclic butyl terephthalate (CBT) to fabricate new thermoplastic composites. Okajima et al. [46] decomposed CFRP wastes using supercritical water and potassium carbonate as a catalyst at a temperature of 400 °C, a pressure of 20 MPa, and a reaction time of 45 min. The rCFs obtained retained 85% of their mechanical strength. Khalil et al. [73] studied 17 supercritical fluids used to decompose thermoset resins. They found that a supercritical mixture of solvent and water could be more efficient in recycling rCFs. Henry et al. [74] showed that a water/ethanol mixture (50/50 vol.%) could obtain cleaner rCFs and higher resin removal efficiency. This enhanced efficiency appeared because the faster decomposition rate reduces the contact time between the resin and CFs, reducing the formation of residues on the surfaces of the rCFs. In addition, Henry and Kim reported that shortening the treatment time could make the surface of rCFs cleaner. They also found that after 120 min of treatment with supercritical water, the resin could be completely removed, and rCFs with a cleaner surface could be obtained [45,74].

Introducing acidic or basic compounds, catalysts, and oxygen to the chemical recycling system could accelerate and improve the removal efficiency of the resin effectively. Liu et al. [44] investigated the decomposition process of CFRPs using water as the reaction medium. The results showed that adding 1 M of sulphuric acid solution accelerated the degradation of the epoxy resin. The rCFs obtained were clean with no cracks or defects, and the average tensile strength was 98.2% of vCFs. Bai et al. [47] used chemical recycling by introducing oxygen into supercritical water. The results showed that the rCFs had comparable strength to vCFs when the epoxy resin decomposition rate was 94–97 wt%. Liu and colleagues also demonstrated that using supercritical fluid water and adding phenol and KOH to the system could improve the dissolution rate of epoxy resin with a removal rate greater than 95%. The recovery of rCFs could reach 95.2% and 100% at 315 °C and 325 °C for recycling for 30 min, respectively, with mechanical and surface properties comparable to those of vCFs [44]. Wang et al. [25] used acetic acid and AlCl_3_ as catalysts at 180 °C to recycle rCFs from cured epoxy composites, with a tensile strength of 97% of vCFs. Das et al. [48] mixed acetic acid and hydrogen peroxide to form peroxyacetic acid as an oxidation method to decompose CFRPs and obtained rCFs with a clean surface and similar tensile strength to vCFs. In addition, this method does not require high temperatures and pressure, reducing the environmental impact.

### 2.3. Thermal Recycling Method

#### 2.3.1. Pyrolysis Method

**Figure 4:**
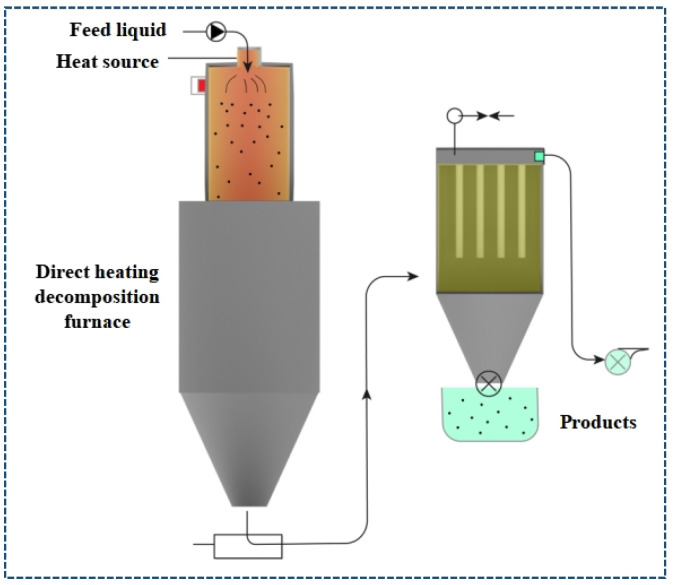
The simplified process diagram of pyrolysis technology.

The basic principle of thermal recycling is to use high temperatures to degrade the polymer matrix and leave a fibrous residue. During pyrolysis, CFRPs decompose after thermal treatment (400–1000 °C) in an oxygen-free atmosphere and produce gases with different molecular weights (e.g., H_2_, CH_4_, and CO_2_), oils (e.g., benzene, toluene, ethylbenzene, and phenols) and solids (CFs and fillers) [75,76]. The simplified process of pyrolysis technology is detailed in Figure 4. Jiang and Rudd et al. [77,78] oxidized the epoxy resin matrix to gaseous products, light aliphatic, and aromatic hydrocarbons at 550 °C, obtaining rCFs that retained 80% of the vCFs. The main advantages of pyrolysis are (1) the energy consumed for recycling rCFs is only 5–10% of that for producing vCFs [17]; (2) rCFs can retain at least 50–75% of mechanical properties, and 90–95% after optimization; and (3) it is possible to implement it on a commercial scale, reaching the industrial scale.

The surface chemistry and mechanical properties of rCFs are highly influenced by the temperature of the thermal recycling process. Too low a temperature results in inadequate substrate degradation, forming an amorphous carbon layer deposited on the rCFs. An auxiliary oxidation process can remove this solid carbon pollution, but toxic waste gas will be discharged. Recently, Curti SpA (Italy) introduced an innovative static bed intermittent pilot reactor [79,80], transforming it into a two-step process: combining pyrolysis and auxiliary oxidation at 500–550 °C. This technology combines the main advantages of both components, saving the energy cost of crushing the feed waste while recovering materials and rCFs can retain up to 95% of their original tensile strength [81]. In addition, too high a temperature helps to reduce the formation of the carbon layer but reduces the fiber thickness. Both cases will cause a significant loss of mechanical properties of the rCFs. However, a new view on such carbon layers was presented by Mazzocchetti et al. [57], who found that the presence of carbon layers could protect the surface of rCFs. The results showed that vCFs were more easily damaged than rCFs in the oxidation process. The reason is that the carbon layer increased the diameter of rCFs to 10% larger than that of vCFs. It could prevent the reaction between oxygen and available carbon atoms in the fiber structure as a protective layer and reduce fibers’ excessive degradation [16,57].

Selecting an appropriate process temperature, oxygen concentration, and reaction time can retain the mechanical properties of rCFs to the maximum extent, which could be adjusted depending on the proportion of gas introduced into the reactor. Nahil and Williams applied different temperatures of 350 °C, 400 °C, 450 °C, 500 °C, and 700 °C during the pyrolysis of braided CF-reinforced polyphenyl oxazine resin (40–45%) and carried out a post-oxidation process at 500 °C and 700 °C, respectively [49]. The results revealed that the pyrolysis and post-oxidation temperatures of 500 °C could maintain the best mechanical properties of rCFs (93% tensile strength and 96% Young’s modulus). In addition, they found that increasing the post-oxidation temperature (500 °C to 700 °C) could shorten the time for removing the carbon layer (2 h to 15 min). However, this also reduced the mechanical properties of rCFs, similar to the findings of Meyer et al. [82]. Yang et al. [50] investigated the effects of oxygen concentration, reaction time, and temperature on the surface and mechanical properties of rCFs. They pyrolyzed 4,4-diaminodiphenylmethane cured epoxy resin under different gas mixtures (100% N_2_, 5% O_2_–95% N_2_, 10% O_2_–90% N_2_, and 100% air), temperatures (550 °C, 600 °C, and 650 °C), and reaction times (15–60 min). The results showed that increasing these parameters could lead to mass loss of rCFs. The main factors influencing this process were the oxygen content and temperature. They increased the oxygen concentration from 5% to 10% and found that the tensile strength of the rCFs decreased rapidly, increasing to 20%, while their tensile modulus decreased. The severe loss of quality and mechanical properties was attributed to the further oxidation of the rCFs after removing the carbon layer. Finally, it was proved that the reaction time of 45 min in the presence of 5% oxygen was the best pyrolysis condition, where the rCFs showed 80% tensile strength retention [16,50]. In summary, the appropriate oxygen content should be considered in the pyrolysis process: it can oxidize the deposited coke and retain its mechanical strength.

#### 2.3.2. Fluidized Bed Method

The fluidized bed process involves chopping the CFRP wastes into a size range of 6–25 mm via a mechanical process and feeding it into a bed of silica sand (size approx. 0.85 mm). The polymer matrix is then separated from the embedded fibers using a gas flow (0.4–1.0 m/s) having a high temperature (400–650 °C) [1]. Compared to other similar processes, the fluidized bed process is more efficient in recycling rCFs and consumes less energy [83]. However, the main disadvantages of this method are (1) the high cost required to maintain a continuous hot air flow [39], (2) the environmental problems associated with the emission of organic solvents and polluting gases, (3) the mechanical properties of rCFs could be seriously degraded, which show only 10–75% of their original tensile strength, and (4) the recovery rate of the polymer is poor. Thus, the reuse of recycled rCFs and polymers is greatly limited.

Since the 21st century, Pickering et al. [84] have developed fluidized bed recycling technology, expanded for nearly 20 years, and is currently an efficient approach at the pilot scale stage. However, there are few articles on this recycling process. Pickering et al. [85] established a commercial-scale fluidized bed factory. The energy required to recover rCFs was 5% lower than that required to produce vCFs, with only an 18.2% loss of tensile strength, while Young’s modulus remained unchanged. In another study, the authors investigated the mechanical properties of rCFs obtained at different fluidized bed process temperatures. The findings showed that the tensile strength of rCFs decreased by about 90% at a temperature of 600 °C. The reason may be the reduction of fiber length and the formation of numerous surface defects. In addition, the hardness of rCFs decreased by 20% at a temperature of 550 °C. Therefore, it is concluded that higher temperatures can rapidly decompose thermosetting polymer, but too high a temperature can significantly degrade fiber [84]. Ming et al. [69] recycled rCFs from CFRP wastes through fluidized bed processes and investigated the impact on energy and the environment. The results showed that the energy requirement for recycling rCFs was much less than vCFs. The authors supposed that further optimization of the fluidized bed recycling process could be necessary to minimize energy utilization and greenhouse gas emissions and improve the economic efficiency and performance of rCFs.

## 3. Recent Optimizations in Recycling Methods

### 3.1. Some Optimizations in the Machining Recycling Method

Since the recovered products are usually used as cost-efficient raw materials, it is necessary to develop optimization technologies to improve product quality and reduce energy consumption. The research shows that for effective mechanical recycling of thermoset composites, the feed material’s thickness and the screen’s size need to be regulated to recycle the product more efficiently and make it suitable for use as a reinforcing material in manufacturing new composites. The size of the waste is first reduced to less than 100 mm using a low-speed crusher and then further ground to a small enough size using a high-speed hammer mill [69]. This optimization approach could finally obtain fine fibers of 2–20 mm in length and polymer particles of less than 100 µm [86].

### 3.2. Pretreatment in a Chemical Recycling Method

Chemical recycling methods using supercritical fluids are an expensive and energy-intensive process. Therefore, some recent research has focused on developing simpler chemical recycling techniques, such as combining pretreatment techniques. This new optimization approach combines oxidants with catalysts, acids, bases, and swelling agents [87]. The function of the pretreatment process is to induce the swell of composite structure and enhance the interaction between catalyst and chemical bonds in resin, facilitating its decomposition [87]. Xu et al. [51] used a two-step process to produce high-quality rCFs from epoxy/carbon fiber (EP/CF) composites. They first pretreated the composites with acetic acid to increase the specific surface area. Subsequently, a synergistic oxidative decomposition system of a mixed solution of N, N-dimethylformamide (DMF), and hydrogen peroxide (H_2_O_2_) was used to recycle rCFs in a closed reactor. The results showed that the decomposition rate of EP was higher than 90% when it was treated in H_2_O_2_/DMF (1:1, *v*/*v*) solution at 90 °C for 30 min, and rCFs with smooth surface and tensile strength higher than 95% were obtained. In addition, strong acid and alkali media are widely used to promote the chemical degradation of resins. For example, Jiang et al. [88] used a mixture of nitric and sulphuric acid as a pretreatment step to decompose CFRPs using a low-temperature and rapid recycling method. The results showed that the decomposition rate of epoxy resin could reach 97% in the presence of potassium hydroxide when treated with polyethylene glycol 400 at 160 °C for 200 min. This way, rCFs with slightly reduced tensile strength (4%) were obtained, and polar groups such as carboxyl and carbonyl groups were introduced to their surfaces.

It was also reported that acetic acid swells CFRPs more easily than alcohol. Wang et al. [25] decomposed the CFRPs under mild conditions using an AlCl_3_/CH_3_COOH system. The results showed that acetic acid effectively swelled the epoxy resin matrix and promoted the selective fracture of C–N bonds in the AlCl_3_, thus achieving 100% removal efficiency of resin. Similarly, H_2_O_2_ could be considered a powerful oxidizing substance with hydroxyl radicals (•OH), efficiently facilitating the degradation process of thermosetting polymers. However, in general, additional heating or catalyst addition is required to improve the degradation efficiency of the resin further. For example, Xu and Wang et al. [25,51] first pretreated CF/epoxy composites with acetic acid at 120 °C to make them swell and better interact with oxidants. The pretreated material was then immersed in an H_2_O_2_/DMF solution and heated using various conditions. In this way, the oxidizing substances produced by H_2_O_2_ could oxidize and decompose organic substances, and the products were dissolved in DMF and separated. Xu et al. [51] reported that the degradation efficiency of the resin was more than 99% at 90 °C, 30 min treatment, and V_DMF_/V_H2O2_ = 1:2. While the rCFs maintained 98% of the tensile strength when treated at 100 °C.

### 3.3. Some Optimizations in the Pyrolysis Recycling Method

#### 3.3.1. Superheated Steam Method

Recently, a new pyrolysis recycling method based on superheated steam (SHS) has been used to obtain high-quality rCFs [89], e.g., using CO_2_ and water vapor to facilitate efficient decarbonization [90]. Wada et al. [91] clarified the effect of SHS treatment on the surface state of the obtained rCFs and their adhesion to the epoxy resin. It was shown that the interfacial shear strength (IFSS) of composites made of rCFs was higher than that of composites based on vCFs at 700 °C in an N_2_ atmosphere. In another study, Kim et al. [89] pyrolyzed CFRP waste in a fixed-bed reactor by the SHS method. The results showed no carbonaceous residue on the surface of the rCFs. Compared with vCFs, the tensile strength and IFSS value of rCFs were 90% and 115%, respectively.

Furthermore, it was shown that FT-IR and XPS found an increased amount of oxygen-containing functional groups after pyrolysis improved the chemical activity between CFs and the resin matrix. Similarly, Jeong et al. [92] successfully recycled rCFs from CFRPs by selecting steam (water) as the oxidizing agent. The SHS pyrolysis efficiently removed the epoxy matrix, and the mechanical properties of the rCFs were adequately retained: the tensile strength was 2.68 GPa (66% of vCFs), and the tensile modulus was 197.05 GPa (100% of vCFs). Enhanced oxygen/nitrogen-containing surface functional groups were also observed, allowing mechanical interfacial strength retention on the fiber surface. Ma et al. [93] reported the positive effect of SHS treatment on the pyrolysis process of CFRPs. The data showed that the tensile strength of rCFs rapidly decreased after high-temperature pyrolysis (to only 50% of the original strength). This may be due to the oxidation process appearing at high temperatures, resulting in numerous defects on the surface of the rCFs. However, after SHS treatment, rCFs had enhanced tensile properties than those treated in the N_2_ atmosphere.

#### 3.3.2. Microwave-Assisted Pyrolysis

**Figure 5:**
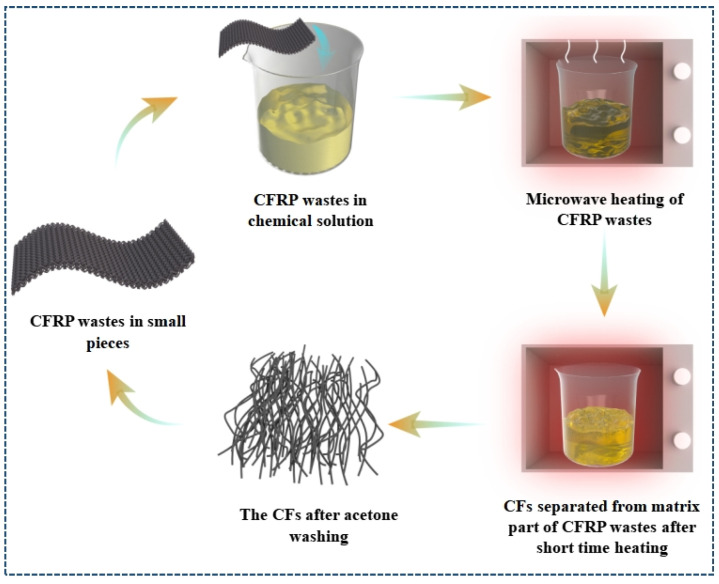
Schematic diagram of chemical recovery of CFRP wastes using MAP technology, Data source: [69].

Microwave-assisted pyrolysis (MAP) uses the energy characteristics of microwaves to heat materials from within by microwaves with ideal selectivity, homogeneity, rapidity, and intrinsic properties [52]. MAP-based techniques have been successfully applied in many areas of organic synthesis and green chemistry [94]. Numerous studies have shown that MAP could offer faster reaction rates, shorter reaction times, higher matrix decomposition rates, higher recovery of rCFs, and lower energy consumption than conventional pyrolysis methods [69]. Figure 5 shows the schematic diagram of the chemical recovery of CFRP wastes using MAP technology. For example, Deng et al. [95] recycled rCFs using MAP and conventional pyrolysis with 94.49% and 93.47% recovery, respectively. Zabihi et al. [96] recovered rCFs from waste composite panels (WCP) in 1–3 min microwave radiation cycle and reported a synergistic effect. The results showed that the decomposition rate of the matrix could reach 95%, while the retention of tensile strength of rCFs was 92%, and the decrease in modulus was negligible. rCFs with clean surfaces and better tensile strength and modulus were recycled from CF-reinforced epoxy composites by Lester et al. [52] using the MAP recycling method (a multi-mode microwave applicator with 3 kW power and a heating time of 8 s). Zhao et al. [97] decomposed the epoxy resin fraction by microwave heating CFRPs and recovered clean rCFs with very similar characteristics to vCFs. Jiang et al. [53] reported the recovery of rCFs from CFRPs using energy-efficient microwave radiation at different temperatures (400 °C, 500 °C, and 600 °C) and determined the optimal irradiation conditions. At lower temperatures, the polymer had more residues but less damage to the fibers than at higher temperatures, so the temperature of 500 °C was chosen as the optimized temperature.

### 3.4. Other Optimizations in the Recycling Methods

Thermoplastics (such as polypropylene, polyamide, and polyethylene) can reversibly transition between plastic and solid depending on the temperature. Recent studies indicate that thermoplastics are being used to recycle CFRPs. For instance, Yamamoto et al. [98] used nylon to recycle high-value CFs from carbon-fiber-reinforced thermoplastics (CFRTPs). The results showed that the modification of CFs by silica-based colloids could improve the interfacial interaction between CFs and nylon, enhance the recyclability of CFs in the nylon-CFRTP matrix, and maintain the mechanical properties of CFs when the nylon matrix is removed. Li et al. [99] studied the mechanism of reinforcing and toughening epoxy resin with three thermoplastic resins (polypropylene-PP, polyamide 6-PA6, and polyether-ether-ketone-PEEK). The results showed that adding thermoplastic resin to epoxy resin could promote the recyclability and reuse of thermoplastic composites. In addition, Butenegro et al. [100] manufactured composites with polyamides (PA11 or PA12) as reinforcement and verified the feasibility of this recycling and reuse route. The study showed that the deterioration of CFs was prevented by the thermoplastic matrix, and the environmental impact was reduced.

Das and Varughese investigated the potential impact of the acoustic chemical recycling process on rCFs [101]. It was shown that the ultrasonic wave in the liquid produces a cavitation phenomenon, and free radical species (e.g., H•, •OH, and HOO•) were generated from the water molecules, increasing the reaction rate. Applying ultrasound and treating CFRPs with diluted nitric acid and hydrogen peroxide solution could enhance the decomposition rate of resin to 95% (three times higher than that without ultrasound treatment), with a tensile strength of rCFs similar to that of vCFs. The authors suggested that investigations could be necessary regarding lowering the ultrasonic treatment time and exploring new solvents to enhance the cost-efficiency of this recycling technique [101]. In addition, Sun et al. [102] proposed a simple, effective, and economical electrochemical method to recycle rCFs. They used NaOH solutions at different current values to determine the optimum recycling efficiency. The results showed that the tensile strength of rCFs could reach 80% of that of vCFs. Zhu et al. [103] proposed a more environmentally friendly and economical recycling method, namely the electrically driven heterogeneous catalysis (EDH), which can recover rCFs at atmospheric pressure and room temperature. The results showed that the tensile strength and IFSS of rCFs were 90% and 121% of that of vCFs, respectively. This method could also increase the commercial value of rCFs and potentially be implemented for large-scale applications. Wu et al. [104] investigated the catalytic pyrolysis of CFRPs in molten zinc chloride at low temperatures (380 °C). They found that molten salts could avoid oxidation of fibers during pyrolysis, and the tensile strength of rCFs reached 95% of vCFs.

## 4. Reuse of rCFs in Photocatalysis

**Figure 6:**
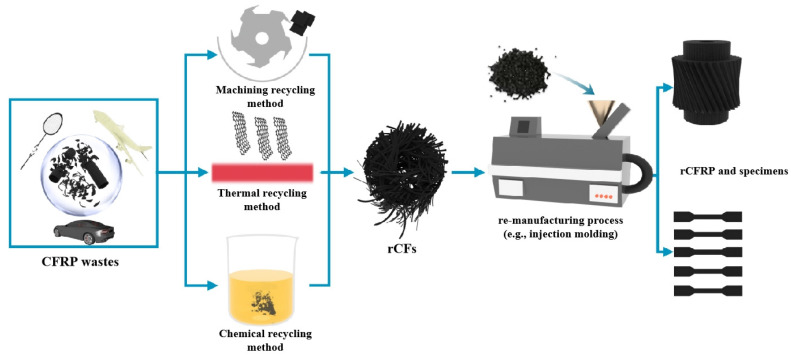
Schematic diagram of recycling methods and re-manufacturing of CFRPs, Data source: [16,109].

To achieve the goal of a circular economy, the reuse of rCFs is a key factor, including the recovery of rCFs from CFRPs or the recycling of dry carbon fiber and the production of new composite materials through compression molding, injection molding, or hot pressing (Figure 6) [39,69,105,106,107]. Reusing rCFs to produce new materials is a part of ongoing investigations. For example, Jagadish et al. [108] reused rCFs to obtain thermoelectric composites with no reduction of conductivity to keep in mind the conductive nature of CFs. Another study demonstrated the feasibility of recycled wind turbine (WT) blades fabricating high-value and high-performance composites through hot pressing technology [107]. In terms of performance, the re-manufacture of composite materials is mainly affected by the physical structure and mechanical properties of rCFs [84]. However, as seen in Section 2 and Section 3, rCFs cannot directly replace vCFs in key structural applications. Some studies discussed that rCFs could damage the combination with new matrix materials [21]. As a result, new CFRPs fabricated using rCFs are still not comparable to commercial CFRPs in some aspects. However, in some potential undeveloped applications, we may be able to use rCFs to improve the performance of new functional materials.

In recent years, many new technologies have been developed to remove pollutants from water or air. The photocatalysis technology based on semiconductor nanomaterials offers a solution for eliminating harmful pollutants. However, the industrial applicability of photocatalysis-based technologies is limited by the separation and loss (photocatalyst itself and performance) of powdered photocatalysts during the recycling process. On the other hand, the composites containing magnetic nanoparticles could effectively separate the powdered photocatalyst from water [110,111]. However, its application in large water bodies is still limited due to the implementation in small containers and the short operating range of the magnetic field.

**Figure 7:**
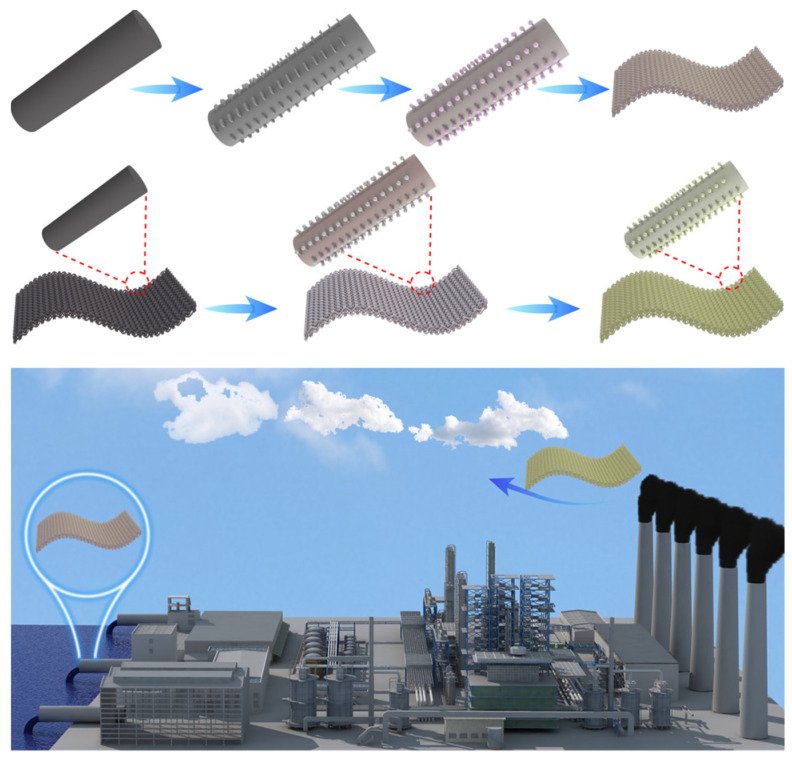
The simplified schematic diagram of semiconductor nanoparticles supported on CFs or CFCs as separable and recyclable photocatalysts for treating wastewater and air pollution.

The research shows that powdered photocatalysts can be deposited on a large area of the substrate surface with a porous structure or rough surfaces, such as carbon materials [112], glass plates [113], zeolites [114], and stainless steel [115]. After the photocatalytic reaction, the substrate can be easily removed from the solution system, so it is a large-scale application. Among them, carbon materials (such as CFs) are very interesting in the structural support and enhancement of photocatalytic performance of semiconductor-based composite photocatalytic systems [116,117,118]. For example, CFs, as carbonaceous materials, could be used to improve the optical properties of semiconductors. It was found that the heterojunction constructed on CFs has practical wastewater/waste gas treatment applications through interface regulation. Figure 7 shows the semiconductor nanoparticles supported on CFs/carbon fiber clothes (CFCs) as separable and recyclable composite photocatalytic systems for treating the flowing wastewater and air pollution.

Thus, rCFs-supported photocatalysis is likely to have a high potential for practical applications in environmental protection. However, no reports have been found in this area, as this new way to reuse rCFs was previously ignored. Therefore, this section reviewed the relevant literature reports on the use of CFs, activated carbon fibers (ACFs), or CFCs to fabricate photocatalytic products for the degradation of environmental pollutants in recent years, giving some inspiration to the research on the recycling of rCFs. Table 2 shows the latest research reports on the CF/CFC/ACF-supported composite photocatalytic systems in environmental purification, including air and water pollution.

### 4.1. Degradation of Dyes

Organic dyes discharged into the environment by industrial activities such as the textile industry, including methylene blue (MB), rhodamine B (RhB), alkaline blue 41 (BB41), and acid orange 7 (AO7), could lead to serious contamination of soil and water sources. The semiconductor photocatalysis technology can completely remove these pollutants from the water environment. Carbon fiber materials could enhance the recovery rate and performance of supported photocatalysts due to their mechanical properties, electrical conductivity, and thermal stability. It was reported that nanoparticles could grow well on CFs. For example, Zhang et al. [119] successfully prepared CuS/ZnO/CFs that are easy to separate and recover by growing CuS/ZnO heterostructure arrays on CFs. Under visible light and simulated sunlight irradiation, CuS/ZnO/CFs showed excellent photocatalytic activity and stability in the degradation of MB. It is mainly due to the effective separation of electron-hole pairs and the coupling effect between the type-II heterostructure of CuS/ZnO and the CFs, improving visible light utilization. Li et al. [120] grew N-doped ordered mesoporous titanium dioxide on CFs (NOMT/CFs). Due to the three synergistic effects of the effective separation of electron-hole pairs, the enhancement of visible light absorption, and the high adsorption of dye molecules, NOMT/CFs showed high photocatalytic activity for AO7 degradation under visible light irradiation with good recyclability. Lv et al. [124] used CFs and TiH_2_ as raw materials to grow TiC on the surface of CFs and then prepared CFs@TiC/TiO_2_ composites. The results showed that CFs@TiC/TiO_2_ positively influenced the photocatalytic degradation of organic pollutants and the photocatalytic reduction of hexavalent chromium [Cr(VI)].

CFCs (reinforced products woven from CFs) have comprehensive performance advantages such as excellent adsorption, conductivity, and mechanical properties (in the case of speed skating skates, CFCs as a shoe shell can make skates lightweight), as well as high flexibility, high porosity, and easy recycling. CFCs provided new prospects for developing flexible filter membrane-based photocatalytic materials. Such materials can offer the advantages of both photocatalysts and filter membranes but without the pollution and high energy consumption problems associated with traditional filter membranes. For example, Chen’s group constructed C_3_N_4_ [126], TiO_2_/C_3_N_4_ [127], C_3_N_4_/BiOBr [122], and TiO_2_/Ag_3_PO_4_ nanostructures [128] on carbon fiber bundles or cloths and successfully applied them for degradation of organic pollutants in wastewaters. Shen et al. [140] constructed C_3_N_4_/CdS nanostructures on CFCs, and the prepared filter membrane-type photocatalysts showed excellent activity (such as 99% MB and 98% AO7) for different pollutants under visible light irradiation, mainly attributed to enhanced light absorption and photocurrent, as well as the separation of photogenerated electron-hole pairs. Behpour et al. [141] prepared a stable and flexible photocatalyst by fixing Fe_2_O_3_/TiO_2_ on CFCs and investigated its activity in degrading the textile dye BB41 under visible light. The results showed that the sample degraded 97.54% of BB41 in 240 min, and only 13% of the photoactivity was lost after 7 reaction cycles (28 h). However, these studies are just starting, and most water pollution purification systems are in the laboratory stage, which is still lacking in large-scale/industrial applicability.

Compared to granular activated carbons, ACFs have more homogeneous micropores, larger pore volumes, adsorption capacities, and faster adsorption rates, thus allowing for good adsorption and separation of organic molecules in aqueous solutions. For example, Kadirova et al. [131] studied the adsorption and photodegradation of MB in oxalate (OA) solutions by Fe_2_O_3_-supported activated carbon felt samples (Fe-ACFTs) under UV light. The results showed almost complete decolorization of MB (96–98%) and confirmed the simultaneous mineralization of MB and OA solutions. Li et al. [132] fixed bismuth halide binary oxide BiOI_0.5_Cl_0.5_ on ACFs to obtain recyclable photocatalysts (ACF@BiOI_0.5_Cl_0.5_). The as-modified material could completely degrade anionic dyes in water through synergistic adsorption and photocatalysis. Luo et al. [133] used sol-gel and hydrothermal methods to obtain well-oriented ZnO nanorod arrays (ZnO NRAs) on ACFs for the photodegradation of MB in an aqueous solution. They found that the synergistic effect between ACFs and ZnO NRAs enabled the composite to exhibit high degradation (77.5%) and mineralization (55.0%) of MB with good reusability. Recently, Tian et al. [142] successfully prepared a Bi_2_O_2_CO_3_/ACF composite photocatalyst by a hydrothermal method, degraded RhB, and removed Cr(VI) under visible light with a three-fold higher performance than Bi_2_O_2_CO_3_, demonstrating its applicability in wastewater treatment.

### 4.2. Degradation of Emerging Pollutants

In recent years, emerging pollutants in modern society, including antibiotics, pesticides, and insecticides derived from animal farming and agriculture, as well as drugs and personal care products, have become one of the main sources of surface water and groundwater pollution. Demircivi et al. [123] used CFs to reduce the band gap of BaTiO_3_ and investigated the photoactivity and stability of BaTiO_3_/CF toward tetracycline (TC) under ultraviolet and visible light. The results showed that BaTiO_3_/CF (5 wt.%) had the highest degradation rate (96%) under UV light. Stability tests showed that the degradation rate of TC was 96% after the first cycle and 77% after the fifth cycle. Furthermore, developing new heterojunction-decorated CFs as wearable photocatalysts could provide an effective strategy for eliminating drug pollutants. In 2020, Zhang et al. [125] proposed the modification of TiO_2_ nanorods on CFs using MIL-101(Fe) nanodots. Subsequently, the CFs/TiO_2_/MIL-101(Fe) bundles were woven into a macroscopic cloth (4 × 4 cm^2^), adsorbing 46.9% of 17b-estradiol (E2) and 40.2% of TC after 60 min in the dark. Importantly, under visible light irradiation, CFs/TiO_2_/MIL-101 (Fe) cloth could remove 87.4% of E2 and 94.2% of TC in 60 min due to its high adsorption and the synergistic photocatalytic effect of three components. In the same year, Shi et al. [122] achieved in situ growth of g-C_3_N_4_/BiOBr heterojunctions on CF bundles by a two-step thermal condensation and chemical deposition method and wove CFs/g-C_3_N_4_/BiOBr bundles into cloths (5 × 5 cm^2^). The results showed that 24 cloths could photocatalytically degrade 86.1% tetracycline hydrochloride (TC-HCl).

Qian et al. [129] reported that a three-component filter membrane photocatalyst (CFC/UiO-66-NH_2_/AgI) could effectively adsorb 19% of levofloxacin (LVFX) or 18.4% ciprofloxacin. Moreover, it could degrade 84.5% LVFX or 79.6% ciprofloxacin (CIP) within 120 min under visible light irradiation. The photocatalytic fuel cell (PFC) could combine photocatalysis and fuel cells. Nahyoon et al. [143] prepared Fe/GTiP anode and ZnIn_2_S_4_ cathode and loaded them on CFCs. In the PFC at pH 7, 0.65 V electric energy was generated using visible light, and up to 79% of the antibiotic-like organic pollutant berberine chloride (BC) was removed in 90 min. Under photocatalysis, Fe/GTiP also achieved 89% TC removal, significantly higher than commercial P25 and pure TiO_2_.

It was reported that Kar et al. [134] constructed heterojunctions (Fe_2_O_3_@BC) on ACFs, which could exhibit excellent photocatalytic activities for the degradation of antibiotics under visible light. Furthermore, Jiang et al. [135] deposited spinel-type CoFe_2_O_4_ on ACFs. Due to the synergistic interaction between CoFe_2_O_4_ and ACFs, ACF/CoFe_2_O_4_ could effectively remove the pesticide pollutant atrazine (ATZ) by adsorption and photo-Fenton degradation. In the work of Dang et al. [136], a titanate nanotube (TNTs@ACF) supported on ACFs was synthesized by a one-step hydrothermal method, applied to the adsorption and photocatalytic degradation of personal care products (PPCP). The results showed that diclofenac (DCF, a typical PPCP) was rapidly adsorbed on the TNTs@ACF, which was photodegraded (98.8%) within 2 h under sunlight. Excellent photocatalytic performance is due to its well-blended structure, allowing TNTs and ACFs to co-adsorb DCF and extending the light absorbance to the visible region.

### 4.3. Antimicrobial Application

To sustainably solve the drinking water safety problem, efficient water disinfection methods are urgently needed. The current disinfection methods mainly use chemical reagents, but the derived disinfection by-products (DBPs) are considered carcinogens, mutagens, or teratogens. Therefore, DBPs free photocatalysts with antibacterial activity could be used as multipurpose disinfectants for drinking water. It has been shown that ACFs deposited with antimicrobial agents not only increase the adsorption of bacteria to the agent but could also show excellent stability and recyclability. However, ACFs adhering to bacteria could become pollutants. For example, Saleem et al. [137] synthesized a novel nanostructured BiVO_4_@ACF by a solvothermal method. Their photocatalytic and antibacterial properties were evaluated using a model contaminant RhB and pathogenic microorganisms (*E. coli* and *S. aureus*). According to the results, the composite material revealed enhanced photocatalytic and antibacterial activity, good chemical stability, and recyclability, demonstrating its potential for water purification and other biological applications.

### 4.4. Degradation of Volatile Pollutants

The air pollutants from industrial facilities and the coal combustion process include volatile organic compounds (VOCs) and nitrogen oxides (NO_x_), which seriously impact human health and the ecological environment. Photocatalytic oxidation (PCO) is an important strategy for removing VOCs. However, the powder photocatalyst could be easily blown away in the continuous gas flow system, resulting in secondary pollution. Some studies have also proved that firmly depositing nanoparticles on the surface of textiles or other fiber materials by immobilization or coating methods could enhance the photocatalytic performance of various air pollutants and make recycling easier. However, so far, only a few reports have discussed the air purification application of such materials.

Kusiak-Nejman et al. [130] prepared AgNPs/TiO_2_ photocatalysts by a wet impregnation method and loaded them on CFCs as additives. The results showed that compared with unmodified CFCs, the composite material was efficient in removing NO from the air (the minimum and maximum NO removal rates were about 80% and 95%, respectively). In 2017, Li et al. [138] prepared nanostructured TiO_2_/ACFF porous composite materials by in-situ depositing TiO_2_ microspheres on activated carbon fiber felt (ACFF). They found that the synergistic effect between TiO_2_ and ACFF could make TiO_2_/ACFF better photodegrade toluene. When the concentration of toluene was lower than 1150 ppm, the adsorption efficiency of TiO_2_/ACFF could reach 98%, and it could also reach 77% at a high concentration of 6900 ppm, avoiding the secondary pollution caused by toluene desorption. Shi et al. [144] first synthesized Au nanoparticle-modified TiO_2_ nanowires (Au/TiO_2_NWs@CFP) ternary composite materials. The composite material showed good photoactivity and photostability to gaseous styrene under visible light irradiation. This is due to the synergistic effect between the three components, improving their adsorption capacity for organic pollutants and reducing the recombination of photogenerated charges. Yao et al. [145] reported a simple surface modification method to enable textile photocatalytic self-cleaning and degradation of indoor volatile organic pollutants. They sprayed graphite carbon nitride nanosheets (CNNs) in colloidal suspension directly on the surface of cellulose fibers to modify the textiles. The results showed that the textile could effectively decompose gaseous formaldehyde using a xenon lamp or commercial LED lamp as a light source.

Carbon dioxide (CO_2_) is the main greenhouse gas in the atmosphere. Due to the excessive dependence of human beings on fossil resources, the increase in CO_2_ emissions is a serious problem. Therefore, the photocatalytic reduction of CO_2_ to clean fuels and energy-rich chemicals has become one of the most attractive solutions to mitigate global warming and the energy crisis. Various photocatalytic materials have been investigated to achieve high activity and selectivity for CO_2_ reduction. Among them, CFs, ACFs, or CFCs with large specific surface areas, good electronic conductivity, and CO_2_ adsorption properties have been proven to be promising support materials. For example, Ding et al. [121] successfully prepared Ag nanoparticles decorated CFs (Ag NPs/CFs) by a simple solution impregnation and ultrasonic treatment. The results showed that Ag NPs/CFs had four times higher photocatalytic activity than pure Ag NPs, and improved the selectivity of CO_2_ conversion to CH_3_OH. Sharma et al. [139] fixed nano TiO_2_ loaded with nickel (Ni) on ACFs to prepare a photocatalytic material. ACF supports provided a high specific surface area and significantly decreased the recombination of photo-generated electron-hole pairs. The results showed that the methanol yield of NiO-TiO_2_/ACF was 755.1 μmol·g^−1^ and 986.3 μmol·g^−1^, respectively, under UV and visible light irradiation within 2 h. In addition, NiO-TiO_2_/ACF was easy to separate and reuse from the reaction medium and showed good stability after multiple uses.

## 5. Conclusions and Future Prospects

In this paper, various recycling technologies of CFRPs (such as mechanical, chemical, and thermal methods) and their influencing factors were reviewed, as well as their effects on the mechanical and surface chemical properties of rCFs. Among them, microwave-assisted pyrolysis has been identified as the most feasible sustainable recycling process to achieve high-quality products and low-energy consumption, showing good potential for large-scale application. The optimization schemes of each recycling method were also explained. Researchers have tried to make the performance of rCFs as close as possible to that of vCFs. However, the current technical level shows that in some key structural applications, rCFs cannot replace vCFs. Furthermore, the main challenges are the low mechanical properties of re-manufactured new CFRP products. On the other hand, developing CF/CFC/ACF-supported recyclable photocatalytic materials targets new investigations. Inspired by this, another focus of this paper is to explore whether rCFs can be reused to fabricate recyclable photocatalysts and apply them to environmental purification. Indeed, rCFs have high reuse potential in developing recyclable photocatalytic products.

Limitations of the different recycling methods still prevent their large-scale applications. Therefore, in the context of circular economy, and from the perspective of developing commercially viable recycling and reuse activities, future research can focus on the following aspects: (i) landfill or incineration of CFRP waste needs to be avoided; (ii) new technologies need to be developed to improve the quality of rCFs, and their scalability should be evaluated economically; for example, further efforts are required in the production of composite materials using rCFs, the adjustment of the interface between the fibers and matrix, and the optimization of the recycling methods; (iii) comprehensively understanding the fiber sources used in CFRP waste to accurately assess the suitability, cost and environmental benefits of the recycled components; (iv) exploring the potential of reusing rCFs to develop hybrid yarns and nonwoven products; and (v) developing new applications of rCFs in other high-value uses, such as photocatalytic applications. Recycling and reusing rCFs is an emerging field and is expected to play an important role in the sustainable development of CFs in the future. 

## Figures and Tables

**Table 1:** Investigations dealing with the main recycling methods.

Recycling Method	Principal Advantaged and Critical Issue	Current Recycling Company	The Properties of rCFs	Recycling Material/Chemical Agents	Experimental Condition	Output	Ref.
Landfilling or incineration	Energy recovery but kTons of CFs are lost; Unfriendly environment	-	-	-	-	-	[1]
Mechanical method	Fast processing speed; High energy consumption and rCFs with poor mechanical properties	Procotex (Belgium); University of Manchester (UK)	rCFs with resin residues and poor mechanical properties (such as shortened lengths and uneven surfaces)	Carbon fiber reinforced (CFR) polyether ether ketone	Electronic equipment + sieving	rCFs with 2–10 mm length and 0.16–2 mm thickness	[40]
				CFR epoxy	Microfine mill	rCFs with 20–100 µm diameter	[41]
				CFR epoxy	Rotating blade with a sieve/ball mill	rCFs with 1–10 mm length and 1–10 µm diameter	[42]
Chemical method	rCFs with good quality; Not highly eco-friendly	Hitachi Chemical; V-Carbon (US)	rCFs with almost unaffected mechanical and physical properties	Subcritical and supercritical alcohols (methanol, ethanol 1-propanol, and acetone)	Alkali catalysts were used as reactive-extraction media, 200–450 °C	Clean rCFs retaining 85–99% of strength compared to vCFs	[43]
				Supercritical methanol, 1-propanol, 2-propanol, 1-butanol, 2-butanol, tert-butanol, acetone, and methyl ethyl ketone	Water as reaction medium, 250 °C	Clean, defect or crack-free rCFs attained a tensile strength of about 98.2% of vCFs	[44]
				Supercritical water	Without any catalysts after optimization, 120 min	99.5% resin removal efficiency	[45]
				Supercritical water	Potassium carbonate as catalysts, 400 °C, 20 MPa, 45 min	70.9% phenolic monomer, 85% strength of clean rCFs compared to vCFs	[46]
				Supercritical water	29 MPa–31 MPa, 430–450 °C, 25 min–35 min	Clean rCFs were almost equal to vCFs	[47]
				Peracetic acid (acetic acid + H_2_O_2_)	65 °C, 4 h	Similar to vCFs	[48]
Thermal method	rCFs with good mechanical and chemical properties; High energy recovery and recovery efficiency of products	Alpha Recyclage Composites (France); Carbon Conversions Inc. (Toyota Tsushon America, US); CFK Valley Stade Recycling GmbH & Co. KG (Germany); Curti SpA (Italy); ELG Carbon Fibre (UK); SGL Automotive Carbon Fibres (US)	rCFs can retain at least 50–75% of mechanical properties, and 90–95% after optimization; When the temperature is too low, the rCF are stiff with poor mechanical properties; When the temperature is too high, the rCF with reduced diameters and mechanical properties	The composites made of woven CFs (55–60%) and polybenzoxazine resin (40–45%)	500 °C for 1 h in a static bed reactor; The post-oxidation process was carried out at 500 °C	93% and 96% of the tensile strength and Young’s modulus were maintained	[49]
				Composite made of 4,4-diaminodiphenylmethane cured epoxy resin	650 °C, 5% oxygen, 45 min	rCF showed 80% retention in tensile strength	[50]
				Waste composite panels	Different ratios of H_2_O_2_/TA (1 to 3), 1–3 min	The matrix decomposition yield of up to 95%, the tensile strength retention of 92%, and negligible reduction in the modulus	[51]
				CF epoxy composites	Multimode microwave applicator with power 3 kW and heating time 8 s	Relatively clean rCFs with better tensile strength and modulus	[52]
				CFRPCs	400 °C, 500 °C and 600 °C	Intermediate temperature was selected as the optimized temperature	[53]

**Table 2:** Latest reports on the applications of CF/CFC/ACF-supported composite photocatalytic systems in environmental purification.

Support	Composite Photocatalytic System	Performance Advantage	Target Pollutant	Photocatalytic Effect	Ref.
CFs	CuS/ZnO/CF heterostructures	Easily separated and recycled with little loss in the photocatalytic activity	Methylene blue (MB)	Degraded up to 98.62% after 120 min	[119]
	NOMT/CFs	The CFs serve to concentrate the pollutant around the active sites, favor electron transfer, and facilitate convenient recycling	Acid orange 7 (AO7) solution	KR and KS were 0.015 mg L^−1^ min^−1^ and 4.26 mg L^−1^ min^−1^, respectively	[120]
	Ag NPs/CFs	The increase of CO_2_ adsorption and the efficient electron transfer to CO_2_ as well as the active site splitting of CO_2_ reduction and H_2_O decomposition	CO_2_ photocatalytic reduction	CH_3_OH production is 0.475 μg/mg h	[121]
	CFs/g-C_3_N_4_/BiOBr bundles	Serve as a flexible, wearable and recyclable photocatalyst	Tetracycline hydrochloride (TC-HCl)	Degraded 86.1% in 120 min	[122]
	BaTiO_3_/CF	CFs were used to decrease the band gap energy of BaTiO_3_	Tetracycline (TC)	Degraded 96% under UV light	[123]
	CFs@TiC/TiO_2_ composite	Easily recycled and reused with good reactivity	RhB and Cr(VI)	Degradation of RhB and reduction of Cr(VI) were about 90% and 80%, respectively	[124]
	CFs/TiO_2_/MIL-101(Fe) cloth	Filter-membrane-shaped photocatalyst with efficient, low-cost and recyclable	Pharmaceutical pollutants	Efficiently adsorbed 46.9% 17b-estradiol (E2) and 40.2% TC after 60 min in the dark	[125]
CFCs	CF/C_3_N_4_ cloth (4 × 4 cm^2^)	Excellent flexibility and strong visible-light absorption at ~450 nm	RhB and parachlorophenol (4-CP)	Degraded 98% RhB in 60 min and 99.3% colorless 4-CP after 120 min of visible-light irradiation	[126]
	CF/TiO_2_/C_3_N_4_ cloth (4 × 4 cm^2^)	Excellent visible photoabsorption (edge: ~450 nm) and improved photocurrent	Various pollutants	Degraded 98% MB after 60 min, 93% AO7 in 100 min, 97% 4-CP in 80 min, 82% TC in 60 min, and 97% Cr(VI) after 90 min	[127]
	CFC/TiO_2_/Ag_3_PO_4_ (4 × 4 cm^2^)	Flexible filter-membrane with high photocatalytic activity	Organic pollutants	Under Vis or UV-Vis light illumination, efficiently degraded phenol (80.6%/89.4%), TC (91.7%/94.2%), RhB (98.4%/99.5%) and AO7 (97.6%/98.3%)	[128]
	CFC/UiO-66-NH_2_/AgI (4 × 4 cm^2^)	Recyclable, high adsorption and photocatalytic capacity	Antibiotics	Degraded 19.0% levofloxacin (LVFX) or 18.4% ciprofloxacin (CIP) in 60 min in the dark and degrade 84.5% LVFX or 79.6% CIP in 120 min under visible light irradiation	[129]
	AgNPs/TiO_2_-loaded CFC composite	Good thermal and photocatalytic stability	Nitric oxide (NO)	The minimal and maximal NO removal rates reached about 80% and 95%, respectively	[130]
ACFs	Fe-ACFTs	The combination of heterogeneous and homogeneous photocatalysis	MB	Almost complete decolorization of MB (96–98%) and more than 91% total organic carbon (TOC) removal were achieved	[131]
	ACF@BiOI_0.5_Cl_0.5_	ACF, as flexible, conductive, and corrosion-resistant supports, were beneficial to the photocatalytic degradation process	Anionic dyes	The maximum adsorption efficiency was about 80% in 70 min	[132]
	ZnO NRAs/ACFs	High surface area and intensive blue, green, and yellow emissions, robust recyclability	MB	Degraded 77.5% MB in 2 h	[133]
	Fe_2_O_3_@BC/ACF	Interfacial tuning of the heterojunction and overall charge carrier separation	Emerging pharmaceutical pollutants	Degradation of antipyrine (60%)	[134]
	ACF/CoFe_2_O_4_ composite	Excellent adsorption ability due to the synergistic effect between CoFe_2_O_4_ nanoparticles and ACF felts	Atrazine (ATZ)	Degradation efficiency was 96% in 240 min	[135]
	TNTs@ACF	Well-defined hybrid structure	Pharmaceuticals and personal care products (PPCPs)	Photodegraded 98.8% diclofenac (DCF) under solar light within 2 h	[136]
	BiVO_4_@ACF	Enhanced photocatalytic and antibacterial activity, chemical stability and good recyclability	(RhB) and pathogenic microbes (Escherichia coli and Staphylococcus aureus)	Degraded 86% RhB and inhibited the growth of both bacteria	[137]
	TiO_2_/activated carbon fiber felt (TiO_2_/ACFF) porous composite	Excellent adsorption and photodegradation properties due to the synergetic effects between the nanostructured TiO_2_ and ACFF	Toluene	At the toluene concentrations of 230 ppm and 460 ppm, the photocatalytic oxidation efficiency of toluene into CO_2_ arrives at 100% and 81.5%, respectively	[138]
	NiO-TiO_2_/ ACF	The ACF support decreased the recombination of photo-generated electron-hole pairs	Photocatalytic reduction of CO_2_ to methanol fuel	The methanol yield in 2 h was 755.1 μmol g^−1^ and 986.3 μmol g^−1^ under UV and visible light irradiation, respectively	[139]

## Data Availability

Not applicable.
